# Determination of Antimicrobial Activity and Resistance to Oxidation of *Moringa peregrina* Seed Oil

**DOI:** 10.3390/molecules17032330

**Published:** 2012-02-24

**Authors:** Stavros Lalas, Olga Gortzi, Vasilios Athanasiadis, John Tsaknis, Ioanna Chinou

**Affiliations:** 1 Department of Food Technology, Technological Educational Institution of Larissa (Karditsa Annex), Terma N. Temponera str., GR-43100, Karditsa, Greece; Email: slalas@teilar.gr (S.L.); vs.athanas@gmail.com (V.A.); 2 Department of Food Technology, Technological Educational Institution of Athens, Agiou Spyridonos str., GR-12210, Egaleo, Athens, Greece; Email: jtsaknis@teiath.gr; 3 Division of Pharmacognosy and Chemistry of Natural Products, Department of Pharmacy, University of Athens, Panepistimiopolis-Zografou, GR-15771, Athens, Greece; Email: ichinou@pharm.uoa.gr

**Keywords:** *Moringa peregrina*, seed oil, antimicrobial activity, resistance to oxidation, bacteria

## Abstract

The antimicrobial activity of the oil extracted with n-hexane from the seeds of *Moringa peregrina* was tested against *Staphylococcus aureus*, *S. epidermidis*, *Pseudomonas aeruginosa*, *Escherichia coli*, *Enterobacter cloacae*, *Klebsiella pneumoniae*, *Candida albicans*, *C. tropicalis* and *C. glabrata*. The oil proved effective against all of the tested microorganisms. Standard antibiotics (netilmycin, 5-flucytocine, intraconazole and 7-amino-4-methylcoumarin-3-acetic acid) were used for comparison. The resistance to oxidation of the extracted seed oil was also determined.

## 1. Introduction

A large number of pharmacological investigations have been directed towards the plant kingdom as a source of therapeutic agents [1]. Some of these investigations were carried out on species of Moringaceae family [2,3]. The Moringaceae family consists of up to 12 species [4] which belong to only one genus called Moringa. Morton [5] reported that the most common species are *Moringa oleifera* (syn. *M. pterygosperma* Gaertn.) and *Moringa peregrina* (forsk) fiori [syn. *M. aptera* Gaertn.; *M. arabica* (Lam.) Pers., *Moringa zeylanica* Sieb.; *Balanus myrepsica* Blackm].

These species, that occur in the Red Sea area, Arabia, and the Indian subcontinent are all slender trees. The most economically valuable species, *M. oleifera*, is now cultivated in all the countries of the tropics. *M. oleifera* seems to be native to dry tropical areas in northwestern India, at the southwestern foot of the Himalayas [6]. *M. peregrina* has a wider range, growing from the Dead Sea area sporadically along the Red Sea coasts to northern Somalia and around the Arabian Peninsula to the mouth of the Persian Gulf [7,8].

Edible oils were extracted where the trees were cultivated, by boiling the seeds with water and collecting the oils from the surface of the water [4]. Moringa oil has been used in skin preparations and ointments since Egyptian times [9]. The bright yellow oil, with a pleasant taste, has been compared in quality with olive oil. The kernel contains 35–50% by weight of oil. Recent studies in Ghana showed that soap made with moringa oil was extremely good [10].

In the rural areas of Sudan the powdered seeds of *M. oleifera* are traditionally utilized for water purification because of their ability to coagulate and cause sentimantation of suspended mud and other materials causing turbidity [2]. During this procedure a decrease of the total bacteria count of the purified water was observed, indicating that the seeds contain substances with antimicrobial activity, and 4-(L-ramnosyloxy) benzyl isothiocyanate was identified as an active antimicrobial agent from *M. oleifera* seeds [3]. While the common species of Moringaceae family, the *Moringa oleifera* (Lam.) has been extensively studied as coagulant, disinfectant, antimicrobial, edible oil, *etc*. [1,2,11,12], *M. peregrina* has been the object of very few studies (to our knowledge only as edible oil by Tsaknis [12]). In this work, the antimicrobial activity and antioxidant stability of the *M. peregrina* seed oil were evaluated.

## 2. Results and Discussion

The results of the antimicrobial activity screening of the oil extracted with *n*-hexane from the seeds of *M. peregrina* and the standard antibiotics are summarized in Table 1. The seed oil of *M. peregrina* appeared active against all studied microorganisms. *Candida glabrata* proved the least resistant (MIC 3.25 mg/mL) while *Candida albicans* was the most resistant (MIC 5.70 mg/mL). At the same time, the standard antibiotics used were active only on certain microorganisms. For example netilmycin and AMCA (7-amino-4-methylcoumarin-3-acetic acid) had no effect on *Candida* spp. Conversely, intraconazole and 5-flucytocine were only active on the three human pathogenic fungi (Table 1).

**Table 1:** Minimal inhibitory concentrations (MIC) of the oil (extracted with *n*-hexane from the seeds of *M. peregrina*) and standard antibiotics on test organisms (in mg/mL).

Microorganism	*M. peregrina* seed oil	Intraconazole	5-Flucytocine	Netilmycin	AMCA
***S. aureus***	3.50 ^a^	-	-	4 × 10^−3^	3 × 10^−3^
***S. epidermidis***	3.35	-	-	4 × 10^−3^	3 × 10^−3^
***P. aeruginosa***	4.38	-	-	8.8 × 10^−3^	3.1 × 10^−3^
***E. cloacae***	4.80	-	-	8 × 10^−3^	4.2 × 10^−3^
***K. pneumoniae***	4.30	-	-	8 × 10^−3^	4.8 × 10^−3^
***E. coli***	4.95	-	-	10 × 10^−3^	5 × 10^−3^
***C. albicans***	5.70	1 × 10^−3^	0.1 × 10^−3^	-	-
***C. tropicalis***	3.30	0.1 × 10^−3^	1 × 10^−3^	-	-
***C. glabrata***	3.25	1 × 10^−3^	10 × 10^−3^	-	-

^a^ Values are means of triplicate determinations.

Spiliotis *et al.* [2] tested the antimicrobial activity of water seed extracts and seed oil of three *M. oleifera *varieties on various microorganisms (including *S. aureus*, *S. epidermidis*, *P. aeruginosa*, *E. coli *and *C. albicans*). The observed activity of some extracts was comparable with most of the preservatives used in their study (ethyl paraben, propyl paraben, sorbic acid and sodium benzoate), however no activity of the oil was observed against the microorganisms tested. This difference can possibly be attributed to the different species (*M. oleifera* instead of *M. peregrina*) or the method of oil extraction (cold press instead of *n*-hexane extraction used in our study).

Suarez *et al.* [11] assessed the antimicrobial activity of seed extracts of *M. oleifera *on various microorganisms including *S. aureus *and *E. coli*. The activity of the extract used in their study appeared with MIC (mg/mL) values of 9–18 and 50 for *S. aureus *and *E. coli*, respectively. Again, these activities are much lower than those presented in our work (3.50 for *S. aureus *and 4.95 for *E. coli*, respectively) for *M. peregrina* seed oil.

The susceptibility to oxidation, as determined by the Rancimat method, proved that *M. peregrina* seed oil could resist up to 10.5 h at 120 °C/15 L/h. Additionally, the oil appeared more resistant to oxidation than extra virgin olive oil (8.9 h). The results are in line with those previously reported by Tsaknis [12].

## 3. Experimental

### 3.1. Plant Material

*Moringa peregrina *seeds were obtained from Saudi Arabia. The seeds were air-dried at room temperature (25 °C) for 1 week.

### 3.2. Oil Extraction

The oil from the seeds was extracted with *n*-hexane using the method described by Tsaknis [12].

### 3.3. Determination of the Susceptibility to Oxidation

The susceptibility to oxidation was determined according to the method described by Tsaknis [12] using a Rancimat 743 (Metrohm Ltd, Herisau, Switzerland). The conditions were set at 120 °C and 20 L/h. The induction period of a sample of extra virgin oil was also used for comparison.

### 3.4. Antimicrobial Bioassay

The antibacterial activities of the extracted oil were determined using the diffusion technique of Bauer-Kirby (disk method) [13], by measuring Minimal Inhibitory Concentration (MIC) (expressed as mg/mL) against two Gram positive bacteria: *Staphylococcus aureus* (ATCC 25923), and *S. epidermidis* (ATCC 12228), and four Gram negative ones: *Pseudomonas aeruginosa* (ATCC 27853), *Escherichia coli* (ATCC 25922), *Enterobacter cloacae* (ATCC 13047) and *Klebsiella pneumoniae* (ATCC 13883), as well as against three human pathogenic fungi *Candida albicans* (ATCC 10231), *C. tropicalis* (ATCC 13801) and *C. glabrata* (ATCC 28838). The standard antibiotics netilmycin (Sanofi, Diagnostics Pasteur), 5-flucytocine (Sanofi), intraconazole (Sanofi) and 7-amino-4-methylcoumarin-3-acetic acid (AMCA) (Molecular Probes, Eugene, OR, USA) were used in order to control the sensitivity of the tested bacteria and fungi. For each experiment, a control disk without oil was used as blind control. All the paper disks had a diameter of 6 mm and were deposited on the surface of the seeded trypticase soy agar Petri dishes. a solution of 1 mg/mL of the oil (6 μL) was placed on the disks. The plates were inoculated with the tested organisms to give a final cell concentration of 10^7^ cell/mL and incubated for 48 h at 37 °C. The fungi were grown on Sabouraud’s agar (Pronadisa, Conda Lab., Madrid, Spain) at 25 °C for 48 h. The experiments were repeated three times and the results (diameters in mm) were expressed as average values.

## 4. Conclusions

In conclusion, the oil from the seeds of *M. peregrina* presented activity against all the microorganisms tested. Its activity appeared higher than those of *M. oleifera* seed extracts (as reported in literature). Further work should be carried out for the isolation and identification of the antimicrobial agent present in *M. peregrina* seed oil. The *M. peregrina* seed oil also proved to be more resistant to oxidation than extra virgin olive oil.
